# Implementation of an Early Communication Intervention for Young Children with Cerebral Palsy Using Single-Subject Research Design

**DOI:** 10.3390/jcm12010232

**Published:** 2022-12-28

**Authors:** Roslyn Ward, Elizabeth Barty, Neville Hennessey, Catherine Elliott, Jane Valentine

**Affiliations:** 1 School of Allied Health, Curtin University, Perth 6102, Australia; 2 Institute of Health Research, University of Notre Dame Australia, Fremantle 6160, Australia; 3 Department of Paediatric Rehabilitation, Perth Children’s Hospital, Perth 6009, Australia; 4 Telethon Kids Institute, Perth 6009, Australia

**Keywords:** cerebral palsy, early intervention, infant vocalisations, infants, single-subject research design

## Abstract

The implementation of an intervention protocol aimed at increasing vocal complexity in three pre-linguistic children with cerebral palsy (two males, starting age 15 months, and one female, starting age 16 months) was evaluated utilising a repeated ABA case series design. The study progressed until the children were 36 months of age. Weekly probes with trained and untrained items were administered across each of three intervention blocks. Successive blocks targeted more advanced protophone production and speech movement patterns, individualised for each participant. Positive treatment effects were seen for all participants in terms of a greater rate of achievement of target protophone categories and speech movement patterns. Tau coefficients for trained items demonstrated overall moderate to large AB phase contrast effect sizes, with limited evidence of generalisation to untrained items. Control items featuring protophones and speech movements not targeted for intervention showed no change across phases for any participant. Our data suggest that emerging speech-production skills in prelinguistic infants with CP can be positively influenced through a multimodal intervention focused on capitalising on early periods of plasticity when language learning is most sensitive.

## 1. Introduction

Research shows as many as 85% of two-year-old children with cerebral palsy (CP) present with communication impairment, with only 10% expected to outgrow their delay by 4 years of age [1]. Early communication difficulties in young children with CP may be associated with sensory, motor and/or cognitive impairment [2] and are predictive of later language difficulties [3,4] that place children at risk of educational and social disadvantage and long-term activity and participation limitations [5].

Recent advances in the early identification of CP have seen the development of specific motor interventions for children less than two years of age [6,7]. These interventions were designed to mitigate the cascading consequences of impairment by capitalising on sensitive periods of neuroplasticity in early development [8,9]. In contrast, no such evidence-base of interventions exist for early communication impairment for children at-risk of CP [10]. This is despite the recognition that children who cross performance thresholds “earlier in life have better outcomes later” [11], p. 1609 and research demonstrating the impact of multi-modal experiences during sensitive periods of early development on later language learning [12]. 

For example, Kuhl and colleagues [9,12] identify the period between 6 months and 12 months of age as a sensitive period for phonetic learning, representing the earliest milestone in language acquisition [13]. The shaping or attunement of early speech perception and production to a child’s native language is highly dependent on the multi-modal and bidirectional communicative exchanges that take place between a parent and infant [14]. Research has shown that infants of caregivers who engage in a high proportion of contingent communicative interactions show greater attunement and produce more mature vocalisations [15], with a direct positive influence on vocabulary development [16,17]. 

Moreover, there is good evidence to show that infant vocalisations provide a significant foundation for speech and language learning, as well as social, emotional, and cognitive abilities [18,19,20]. Infant vocalisations considered precursors to speech are termed protophones [21]. They follow a developmental trajectory of increasing vocal control and complexity [17]. For example, infants progress from pre-canonical vocalisations such as marginal babbling, containing consonant-like (closant) and vowel-like (vocant) elements with slow movement transitions, through to the canonical babble stage that features speech-like consonant–vowel (CV) syllables with quick transitions [22]. Whilst the age of emergence varies, it is typically reported infants gain control of basic canonical syllables between 5 and 10 months of age [21]. The canonical babbling stage progresses to more advanced or motorically complex forms and provides a foundation for the child to produce their first words with communicative intent. From this perspective, delays or restrictions in the development of infant vocalizations due to an underlying deficit should be predictive of ongoing constraints on the expansion of these vocal production skills into intelligible language, hence, contributing to communication impairment. Consistent with this expectation, research has shown that delayed emergence of canonical syllables predicts poor expressive language, particularly, vocabulary development, in children [18,20]. Furthermore, recent research focusing on identifying biomarkers in infant vocalisations [23,24] has been undertaken in children with neurodevelopmental conditions, such as autism [25] and Down Syndrome [26], contributing to the development of targeted early interventions designed to ameliorate the impact of communication impairment. The development of infant vocalisations in children with CP with the potential to identify communication impairment and intervene early has received limited attention. 

In 1999, Levin [27] reported on the vocalisations of eight, 12-month-old infants with CP. The babbling of all eight participants was limited to monosyllables with the phonetic repertoire comprised largely of back vowels, plosives and velars. These vocal behaviors were associated with limited oral motor control that included the speech subsystems of respiration, phonation and articulation. Nyman and Lohmander [28] also reported on the canonical babble in three children with CP, representing a subset of 18 children with neurodevelopmental disability. They identified children with CP presented with significantly lower levels of canonical babble and limited phonetic repertoire. More recently, Ward et al. [29] reported longitudinal data of 18 infants with CP, as compared to TD infants, utilising the Infant Monitor of vocal Production (IMP). They identified divergence from typical development in the vocalisations of infants with CP at 9 and 12 months of age suggesting delays in the transition from the pre-canonical to canonical babble stage. Collectively, these findings provide evidence of impaired emergence of speech motor control in very young children with CP, and represent an opportunity for the development of CP specific early interventions to benefit their early speech production skills and subsequent communication development.

Currently, a multi-modal approach to very early intervention is recommended [30]. This includes supporting the social foundations of communication (i.e., joint attention for engagement, and play); building comprehension to facilitate the transition to spoken language [31]; and providing access to expressive communication including building speech production, all embedded in the child’s routine to increase opportunities of practice of targeted skills [32]. These principles are consistent with research showing interventions that target parent–child interactions benefit the development of expressive language skills in children at risk of language impairment [33]. However, few interventions to date have been directed at the development of early vocalisations in infants at risk of motor-speech impairments including infants at risk of CP [34,35,36].

In light of the above, this paper reports on a multi-modal case-series intervention for children under 24 months with communication impairment associated with CP. Consideration was given to recommendations for principles of early communication intervention [33], including CP specific early intervention [10], and theoretical constructs that consider a child’s development to arise from bi-directional interactions within the physical, social, cognitive and environmental domains. 

The PROMPT approach we adopted in the present study encompasses each of the aforementioned elements [37]. PROMPT has previously been used in children with CP [38] and found to be effective in improving speech motor control and intelligibility in children aged 3 years to 14 years. It is an empirically supported and manualised approach guided by key tenets of Dynamic Systems Theory, as illustrated within the PROMPT conceptual framework [37]. Clinicians undertake a dynamic assessment of the physical–sensory, cognitive linguistic and social emotional domains utilising the Global Domain Evaluation to determine intervention goals and priorities for functional communication. This is based on the presumption that “all domains interact during communication and that audition and somatosensory information are equally important in the development and organisation of motor-speech behaviour” [37], p. 477. PROMPT trained clinicians will “alternate” their treatment priorities between the communication domains, with the first intervention priority chosen to achieve the greatest shift. For example, intervention with a child who is pre-linguistic and not engaging in reciprocity, will focus on the social–emotional domain, as their priority. All intervention goals and objectives are functionally motivated and developed with consideration given to the child’s and family’s environment, and sufficient opportunity for repetition and practice within the daily routine.

Within the physical–sensory domain, three intervention priorities are determined using the Systems Analysis Observation and Motor Speech Hierarchy (MSH) [37]. The MSH represents seven stages of motor-speech subsystem control and based upon the inter-hierarchical sequence of motor-speech development [39]. 

A PROMPT session must include tactile input that is used to (a) create an interactive awareness for communication with intention; (b) provide associative mapping for cognitive-linguistic input; and/or (c) develop speech subsystems at the sound, word, or phrase level. In addition to behavioural based studies that have demonstrated modifications to the speech system brought about through tactile input [40], more recent exploratory work by Fiori et al. [41] has identified neural changes in participants subsequent to intervention. 

In summary, this study tests the hypothesis that intervention started before 2 years of age, framed within the PROMPT approach and utilising tactile input, will improve the vocal complexity of children with communication impairment secondary to CP. The single-subject experimental methodology was selected to (a) demonstrate proof-of-concept for a multi-modal intervention, focused on speech sound practice for young children at risk of communication impairment secondary to CP; (b) inform a larger scale research design; and (c) accommodate the heterogeneity of the participants.

Three intervention blocks, each using an ABA sequence, were designed to build successive complexity as follows. Block one focused primarily on preparing the child for learning by building social interaction and reciprocity, teaching targeted words within home-based daily routines and play activities. The tactile input was timed to precede or follow the turn-taking routine, avoiding disruption to the reciprocity of the interaction. It was hypothesised the tactile input during block one intervention would contribute to achieving the vocal production priorities developed for each child, our primary outcome of interest, although these changes may be minimal or not consistently sustained. 

In contrast, block two and three focused more directly on building the complexity and diversity of vocalisations/speech produced with communicative intent and increasing motor control, in accordance with the developed motor-speech priorities for each child in each block. The tactile input was provided to shape articulator (i.e., motor) movements during speech production. It was hypothesised that intervention blocks two and three would be associated with an increase in the use of target vocal patterns or protophones (e.g., CV syllable production) and associated speech movements (e.g., closed to open and open to closed jaw transitions with phonation control) during vocal elicitation tasks. 

Each intervention block included three word sets that targeted increasing motor control, as based on the MSH. Word set 1 contained words with the lowest level of complexity, targeted intervention priority one and were trained throughout the whole intervention phase. As Word set 2 contained a higher level of complexity, these were introduced half-way through the intervention phase to allow initial focus on the priority one word set. Word set 3 represented intervention priority three. These words acted as a control and were not actively trained but were embedded within meaningful daily interactions. 

It was hypothesised a treatment effect would be observed for trained items that were part of the intervention. Untrained items, that is, different words containing the same target protophones and requiring the same speech movement pattern, were included in the elicitation tasks to test for generalisation effects to other items. The third word set represents the control goal and as such no treatment effect was expected.

## 2. Method

### 2.1. Research Design

A single-subject multiple-probe research design with three participants was conducted in compliance with the Single-Case Reporting Guideline in Behavioural Interventions (SCRIBE) Statement [42], and design standards described by Kratochwill et al. [43]. This involved (a) systematic manipulation of the independent variable; (b) systematic measurement of dependent variables by more than one blinded assessor; (c) replication of the study design across phases and participants to demonstrate an intervention effect and experimental control; and (d) a minimum of three data points during each pre-intervention baseline phase.

The study design involved repeated ABA phase sequences with the start of each subsequent intervention (B) phase targeting more complex vocalisations (i.e., AB_1_A, AB_2_A, AB_3_A). The length of the first baseline (A) phase in each ABA sequence ranged from 3 to 4 weeks. This was followed by a 10-week intervention phase and then a 3 to 4 week post intervention baseline phase, also involving no treatment being delivered (A). There were three ABA phase sequences for two participants (P1 and P2) and two sequences for one participant (P3). The third intervention ABA block was not offered to P3 due to the COVID-19 pandemic [44]. 

### 2.2. Participants

Three infants (P1, P2, and P3) with CP were recruited through the ‘at-risk’ early intervention service at Perth Children’s Hospital. The tertiary service adheres to recommendations for early diagnosis [45] and evidence-based practice principles.

Inclusion criteria were: identified as at high-risk of CP at less than 6 months of age, and enrolment in the It Takes Two to Talk (ITTT)—The Hanen Program^®^ (Toronto, ON, Canada) [46] administered through the early intervention service at PCH at or by 12 months of age. The Hanen ITTT Program^®^ is based on best practice principles of building parent–child interactions, utilising daily routines, and establishing a shared expectation of parent implemented intervention through joint planning and coaching [47].

Exclusion criteria were: English not spoken in the home, medically unstable, cortical visual impairment and uncorrected hearing impairment with thresholds greater than 25 dB.

Two participants (P1 and P2) formed a subset of data collected within a larger study, focused on profiling the longitudinal development of communication in young children at-risk of CP [29]. Both P1 and P2 were male and aged 15 months at the start of the present study. P3, a female, was referred into the study at 16 months by their managing speech pathologist, following completion of the Hanen ITTT Program^®^ and a multi-site clinical trial for infants with hemiparesis. All parents completed all sessions of the Hanen ITTT Program^®^.

Table 1 shows the participant characteristics at baseline. All three were at the prelinguistic stage of language development at the onset of this study. Table 2 shows communication status, as measured at baseline across each of the study phases.

### 2.3. Setting

This research study was conducted through the state-wide tertiary rehabilitation service at Perth Children’s Hospital [29,48]. The study was framed within the integrated knowledge-to-action framework [49] and designed to transfer knowledge gained through the study directly into the clinical service. 

The study phases were conducted within the family home. Home visits were conducted on a weekly basis and administered in collaboration with a primary caregiver, primarily the mother. Inclusion of fathers and grandparents took place on an ad hoc basis, around availability for P2 and P3. 

### 2.4. Measures

#### 2.4.1. Baseline Assessments

The following measures were used to assess each child prior to each pre-intervention baseline period.

**The Receptive-Expressive Emergent Language Test-3 (REEL-3)**. The REEL-3 is a standardised assessment of emerging language in children from birth to 3 years of age. Information is obtained through parent interview. Raw scores from the receptive and expressive language scales were converted to standard ability scores (*M* = 100, *SD* = 15) with percentile ranks, and a combined language ability standard score was also obtained. The REEL-3 has been identified as a reference standard for early language assessment [50], with established psychometric properties [51]. In addition, Rome-Flanders and Cronk [52] report longitudinal stability, with predictive validity of later testing results at 15 months and 18 months.

**The Communication and Symbolic Behaviour Scales Developmental Profile (CSBS DP).** The Communication and Symbolic Play Scales (CSBS) DP Behaviour Sample [53] is a standardised measure of early communication and symbolic skills for children 6 months to 2 years of age. Information is obtained through the administration of standardised behavioural sampling that includes communicative temptations, books, construction, and symbolic play. The behaviour sample derives a composite score (*M* = 100, *SD* = 15) from six cluster scores (*M* = 10, *SD* = 3): Communication Function, Communication Means Gestural, Communication Means Vocal, Communication Means Verbal, Reciprocity, and Social-Affective Signalling. Reliability and validity are reported to be high, with the three composite scores a significant predictor of receptive and receptive language outcomes [54].

#### 2.4.2. Dependent Variables

The two primary outcome measures reported in this study are the number of probe items (i.e., words within each word set) produced with communicative intent [55] showing (a) achievement of the targeted protophone category [21], and (b) achievement of the targeted *motor-speech movement pattern* (e.g., bilabial closing and opening gesture) reflecting emergence of speech motor control. These measures were extracted via weekly probes administered in the day of each session in each study phase. 

**Speech Probes**. A wordbook containing personalised pictures/photos representing the individual target words for each intervention priority, was developed for each child. For example, the probe word “bye” requires production of the target CV protophone, as well as a bilabial closing and opening speech movement pattern. Expressive speech probes were selected based on the intervention priorities and the MSH, daily routines and play interests, family relevance and communicative functions, including social words, requesting, nouns, action words, and pronouns [35]. 

The Appendix A provides the word sets for each participant across the intervention blocks. Target words for each participant were selected prior to the commencement of each intervention block and divided into three groups based on motor-speech control as described in the systems analysis observation and the MSH. Word set 1 and 2 both contained trained and untrained words based on the increasing complexity of the MSH represented in priorities one and two, respectively. An equal number of trained to untrained words were allocated a priori to each word set. However, a small number of words (no greater than 3 per participant), were re-allocated during the intervention phase in response to participant interest and motivation (see Appendix A). 

Word set 3 contained control words based on intervention priority three. These words were not targeted and acted as a control probe condition throughout the study. 

### 2.5. Procedure

Approval for this study was obtained from the Child and Adolescent Health Services Ethics Committee (study number 2015221). In addition to the intervention being conducted within the family home, parents agreed to an all-day recording of their infant’s vocalisations up to two times a week, as captured through the Language Environmental Analysis (LENA) Digital Language Processor (DLP).

**Figure 1:**
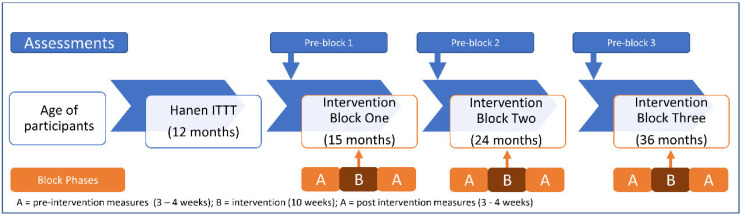
Study phases and timeline.

All participants completed standardised assessments of communication within a 2 week period prior to the commencement of each intervention block. Standards assessments took place within the family home, administered by the first author. Following completion and scoring of the standardised assessments, a second home visit was conducted within 7 days to discuss the assessment results and in collaboration with the family determined the intervention goals, priorities and vocabulary (trained, untrained and control) for each intervention block. Figure 1 illustrates the study phases and timepoints. The speech sample obtained during administration of the CSBS was used to inform the three motor-speech priorities targeted in each intervention block. All sessions in each phase of the study were video recorded (Sony Handycam HDR-CX405). 

#### Administration of Speech Probes

**Baseline phase**. Weekly home visits were conducted to administer the expressive speech probes. Participants were offered a maximum of five opportunities to elicit the targeted verbal response. The planned elicitation strategy was a mand-model (e.g., say X), or open ended prompt (e.g., holding the picture or object represented in the picture book and asking “this is a ?”). However, upon commencement of baseline for intervention block one, it became clear not all participants could respond to these elicitation strategies. As a result, a hierarchy of elicitation procedures was developed and included: cloze with time delay when the word was elicited through a familiar nursery rhyme (e.g., row, row, row your …) and information (e.g., use your lips, /b/). When participants attempted multiple repetitions and self-correction, the best of the first two trials was scored. The elicitation task took between 10 and 20 min to complete for each child, with the order of presentation varied.

**Intervention phase**. Following completion of each home-based therapy session across all intervention blocks, participants were presented with their books to elicit the target word, using the same procedure as the baseline sessions. The exception to this was the speech probes for P2 in block one. P2 was unable to complete the speech probes at the end of the intervention sessions in block one, therefore, the parent was asked to elicit the probes on a selected day at the same time during the week whilst wearing the LENA device. With the investigator given permission to extract the audio file, the parents were asked to identify a period in the day where the speech probes were elicited. The extracted wav files were then used for analysis. The speech movement targets that rely on visual as well as audio information could not be scored during that intervention block. 

### 2.6. Scoring of Speech Probes

Video recordings of the speech probes were converted to wav files, exported to Praat software [56] and visually inspected using the time-amplitude waveform and wide-band spectrograms. Onsets and offsets for each of the target words were annotated in a Praat textgrid and coded using broad phonetic transcription ready for scoring. Three independent PROMPT trained speech-language pathologists (referred to as raters), blinded to the ages of the child, phases of the study, intervention blocks and intervention objectives, completed the scoring of the dependent variables. The individual sessions and target words were randomised during scoring, using the Excel random function. 

**Protophone coding**. Rater 1, who coded the elicited vocal productions, has a master’s degree in linguistics and more than 30 years paediatric clinical experience working with neurodevelopmental disorders, including CP; as well as research experience in the coding of protophones according to Stark protocol [21]. 

The operational definitions used to code the protophones of the vocalisations/word approximations elicited were based on the Stark Assessment of Early Vocal Development-Revised (SAEDV-R) [21]. A binary scoring (1 or 0) was used where the vocalisation was scored for the presence (i.e., achievement) (1) or absence (score = 0) of the targeted protophone for each target word for each participant, with a maximum score of 1 allocated to each word.

**Speech movement patterns (speech motor control)**. Raters 2 and 3 coded the speech movement patterns of elicited vocal productions of the participants. Rater 2 has worked clinically as a PROMPT trained clinician for 20+ years, with a clinical caseload that includes children with motor-speech disorders, including CP. In addition, rater 2 has experience coding speech movement patterns as a research assistant. Rater 3 is a speech-language pathologist with 8 years clinical experience in the assessment and management of speech sound disorders, including CP. She has been trained in the scoring the Motor Speech Hierarchy Probe Words [57]. Similar to protophone scoring the targeted movement pattern was then scored from the digital video recordings for the presence (1) or absence (0) of the identified speech movement pattern for each word, with a maximum score of 1 allocated, with the most accurate production of the first two vocal attempts selected.

**Reliability**. Inter-rater agreement for protophone coding was assessed by the first author and rater 1 both scoring a separate data set, with the amount equivalent to 10% of the coding of the present study, to ensure independence from the data analysis. Good levels of agreement in both percentage (89%) and correlation for agreement using Cohen’s kappa (0.864, *p* < 0.001) was obtained (Hartman et al., 2004).

Good level of inter-rater agreement using Cohen’s kappa of speech movement coding between rater 2 and rater 3, calculated on 10% of the data, was achieved, K = 0.681 (95% CI, 0.600 to 0.748), *p* < 0.001. 

#### Intervention Protocol

Intervention priorities were selected to address the social–emotional, cognitive-linguistic and physical–sensory domains, for each child, as represented within the PROMPT conceptual framework [37]. The intervention routines were developed in consultation with the family and targeted the following three activities: daily routine/play activity, social routine (songs and nursery rhymes), and interactive book-share. Therapy routines were established to allow children to anticipate the targeted vocabulary. For example, cloze techniques during song routines (e.g., “row, row, row your … [boat])” and activities (e.g., stacking cups “up” when placing cups on top of each other).

Block one prioritised turn-taking within parent–child interactions. Activities at the cognitive-linguistic level included building spatial concepts and following single stage instructions. Linguistic input was supported by key word signs and picture supports, as required by the child. The aim was to increase comprehension of salient and meaningful vocabulary for active participation in daily routines and activities. Additionally, the physical–sensory domain of block one informed the three articulatory subsystem priorities that were identified using the MSH and systems analysis observation, as based on the CSBS DP speech sample administered during the pre-baseline assessments. 

In blocks two and three, the social routines established in block one were extended or modified in keeping with the child’s interests. Linguistic input continued to be supported by key word signs and picture supports, as required by the child. Within the physical–sensory domain, three articulatory subsystem priorities were identified using the MSH and systems analysis observation, as based on the CSBS DP speech sample administered in the pre-baseline assessments, as well as the level of success achieved in the preceding block. The emphasis on speech subsystem organisation was increased with the introduction of the motor phoneme warm-up at the commencement of each therapy session. Tactile input was used to facilitate the formation of sensory-motor pathways for speech production. 

Table 3 details the intervention goals for each participant across the study phases. For P1 and P3, intervention blocks one and two focused on refining objectives within the same levels of the MSH. Intervention block three also targeted increased motor complexity at a higher level of the MSH. For P2, the treatment priorities established in intervention block one were further refined in treatment blocks two and three with a focused on increasing the accuracy and variability in syllable structure. 

Participants received therapy once a week for a duration of approximately 45 min. The first 5–10 min were spent in parent discussion reviewing intervention goals and home practice during the week, followed by 30 min active therapy with parent coaching, and the last 10 min were spent planning implementation within the daily routine. The speech probe elicitations for that session were then carried out. The therapy format was individualised to each participant, with the same format followed throughout the intervention block.

**Intervention fidelity**. Intervention fidelity was secured for all participants through the delivery of the intervention protocols by a certified to fidelity PROMPT Instructor (RW), who also has 30+ years’ clinical experience. The instructor has collaborated with Ms Deborah Hayden (PROMPT founder and research director) in previous research protocols [38,58], as well as validation of the PROMPT fidelity checklist [59] and Motor-Speech Hierarchy-Probe Words scoring system [60], and ongoing development of the PROMPT approach to intervention [37]. RW prepared the data for analysis but did not contribute to the scoring of the data.

**Procedural fidelity: dosage**. Fidelity to intervention intensity, as described by Warren, Fey and Yoder [61], was recorded and extracted for 50% of the intervention sessions. Table 4 illustrates the total number of intervention sessions attended, with the average therapy duration and dosage of the active ingredient based on the analysed sessions. Dosage of the active ingredient includes a count of the teaching episodes with tactile input, per minute, where the child was actively engaged in a play routine. Furthermore, the proportion of word set 1 and word set 2 words trained, was calculated. Our data show the active treatment ingredient was administered at more than 1 teaching episode per minute for all participants, except for P3 block one.

### 2.7. Analyses

Visual inspection was undertaken to determine evidence of a relationship between the independent variable (intervention) and the dependent variable (outcome measures). Within and between-phase data patterns were evaluated for change in magnitude (level), trend (direction of performance), variability (degree of overall scatter) between the study phases and consistency of data patterns across the study phases.

Visual analysis was supplemented with the nonparametric Tau-U analysis to determine statistically significant change. Tau-U measures nonoverlap between pre-intervention baseline and intervention phases, and provides a non-parametric Tau coefficient (varies between −1 and 1) to yield effect size estimates [62]. The following Tau benchmarks were applied to document treatment effects: <0.20 small, 0.20 to <0.60 moderate, 0.60 to <0.80 large, and >0.80 very large [63]. A Tau-U phase contrast *p* value < 0.1, where Tau was positive (i.e., 0.05 one-tailed probability test equivalent), was considered statistically significant. 

Finally, the numerical difference between the mean of the post-intervention baseline values, expressed as a percentage of items within the corresponding word set, and pre-intervention baseline percentage values for each ABA time series was calculated to capture the increase in level of performance (i.e., percentage increase in the number of achievements of the target vocalisation pattern) after the intervention stopped relative to the pre-intervention baseline. 

## 3. Results

**Figure 2:**
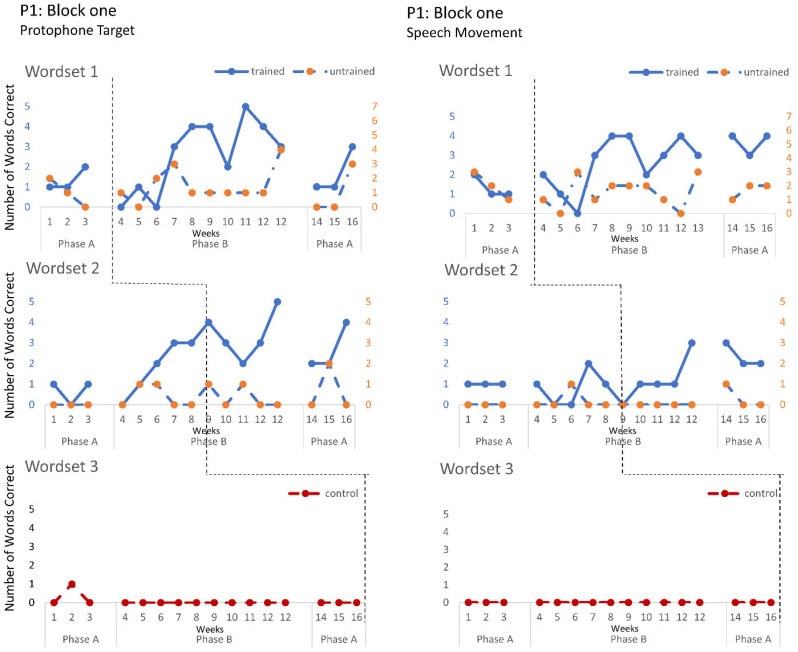
Accuracy of performance on the speech probes as scored for protophone target (**left**) and motor-speech movements (**right**) block one, participant 1.

**Figure 3:**
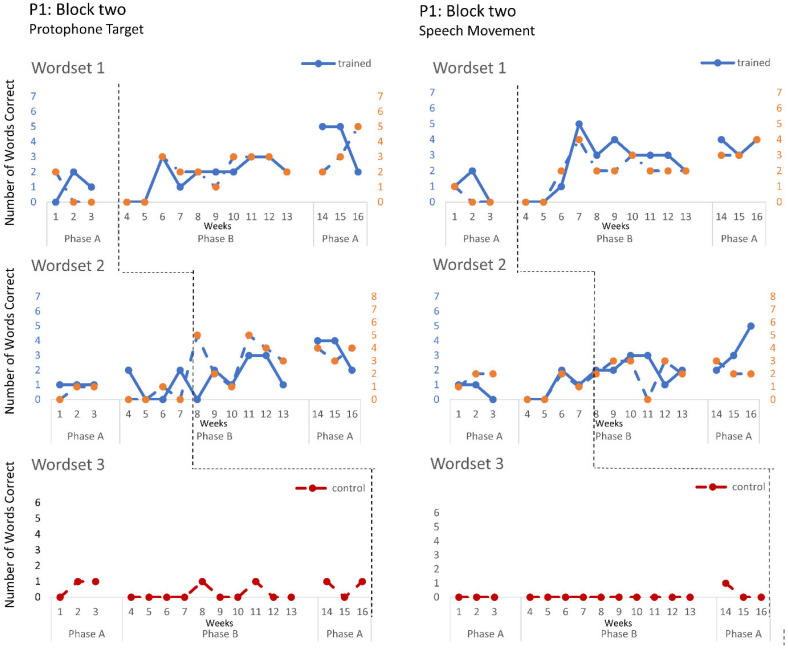
Accuracy of performance on the speech probes as scored for protophone target (**left**) and motor-speech movements (**right**) block two, participant 1.

**Figure 4:**
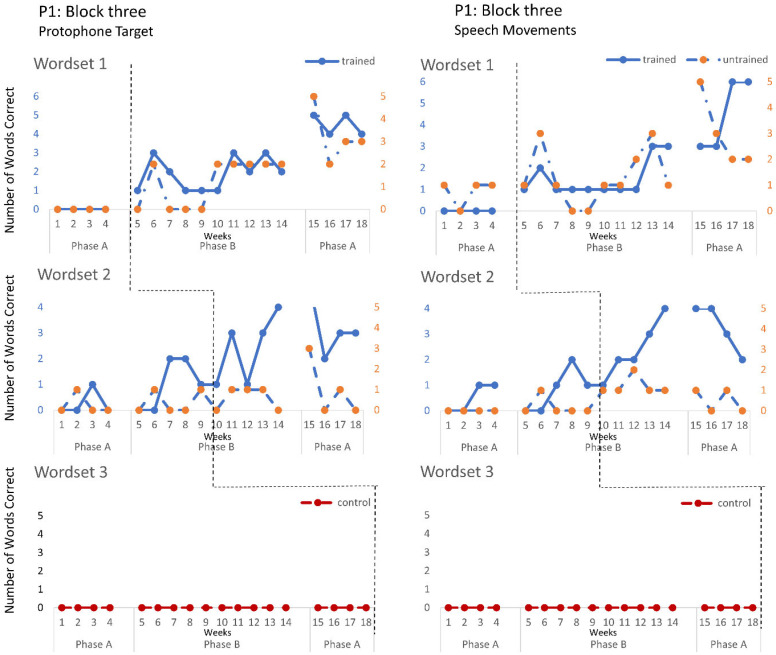
Accuracy of performance on the speech probes as scored for protophone target (**left**) and motor-speech movements (**right**) block three, participant 1.

**Figure 5:**
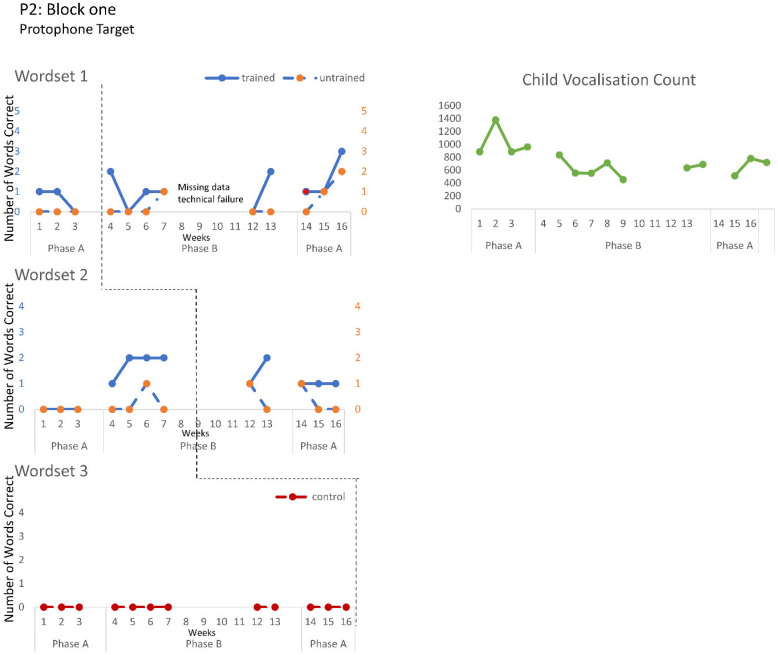
Accuracy of performance on the speech probes as scored for protophone target block one, participant 2 (vocalisation counts obtained from LENA device over same periods as block one).

**Figure 6:**
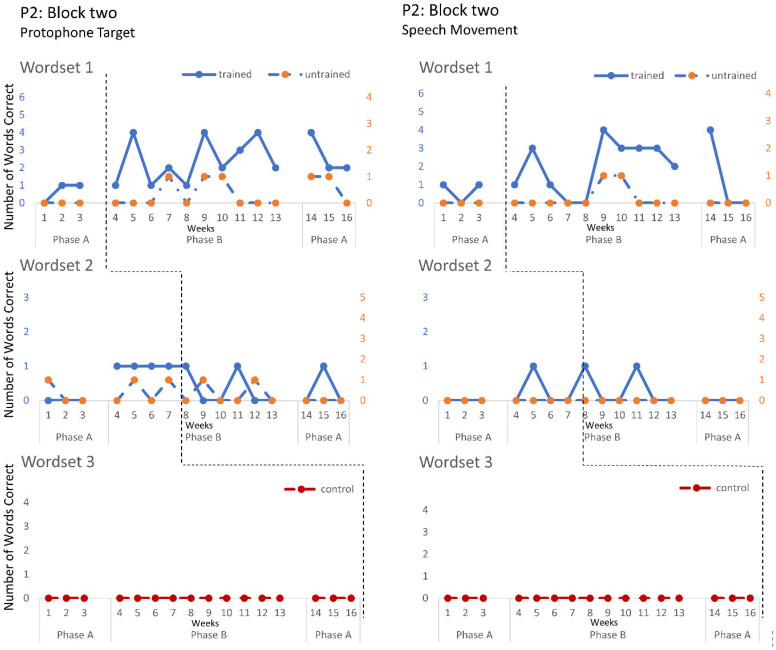
Accuracy of performance on the speech probes as scored for protophone target (**left**) and motor-speech movements (**right**) block two, participant 2.

**Figure 7:**
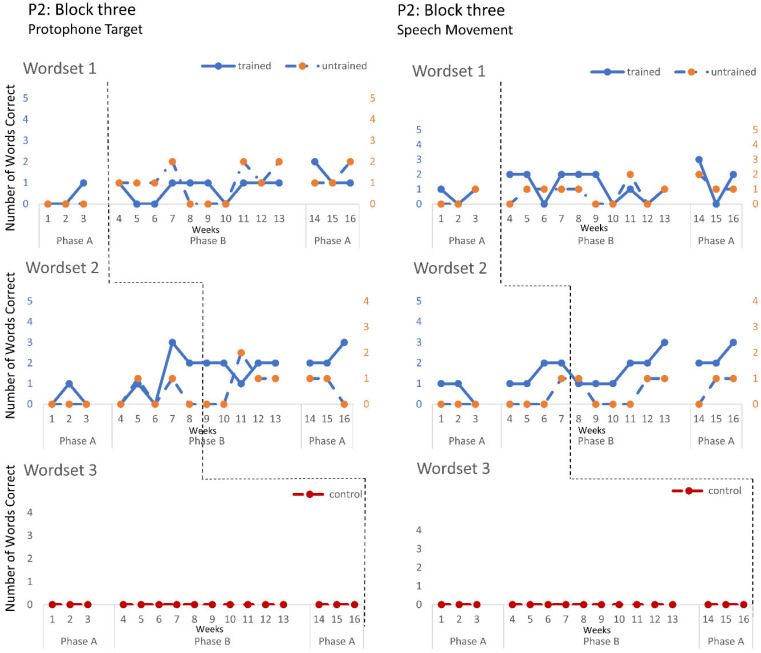
Accuracy of performance on the speech probes as scored for protophone target (**left**) and motor-speech movements (**right**) block three, participant 2.

**Figure 8:**
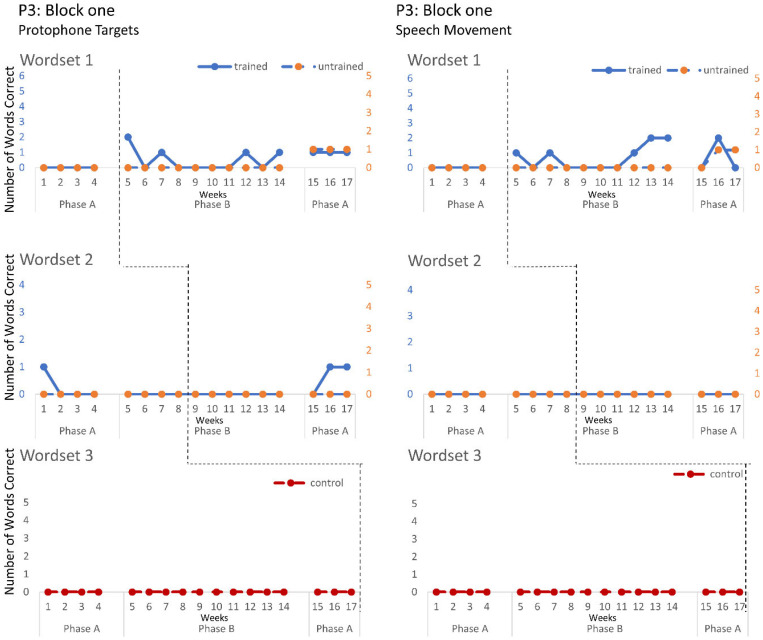
Accuracy of performance on the speech probes as scored for protophone target (**left**) and motor-speech movements (**right**) block one, participant 3.

**Figure 9:**
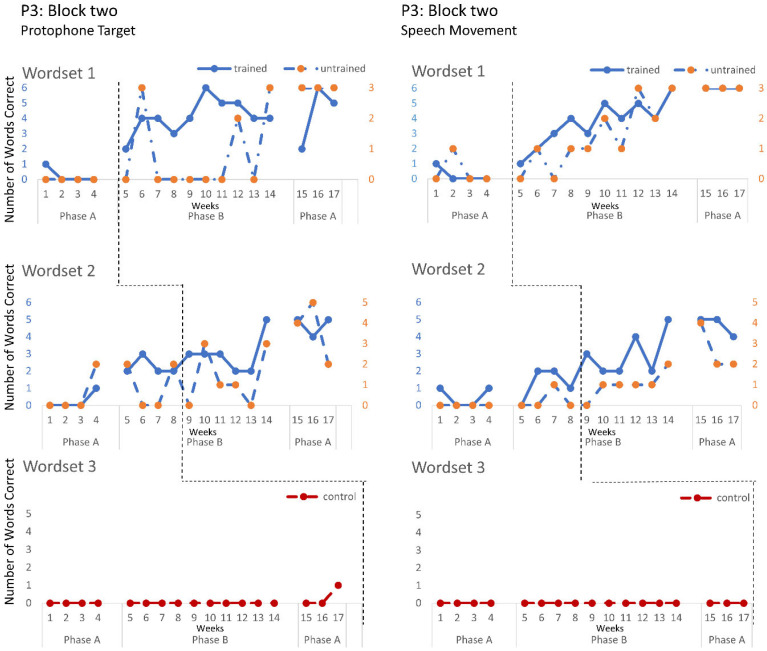
Accuracy of performance on the speech probes as scored for protophone target (**left**) and motor-speech movements (**right**) block two, participant 3.

The speech probe data plotted across the study phases for each intervention block are shown in Figure 2, Figure 3 and Figure 4 for P1, Figure 5, Figure 6 and Figure 7 for P2, and Figure 8 and Figure 9 for P3. Each data point represents the number of elicited vocal productions coded as achieved within each word set for protophone targets (in panel A) and speech movement targets (in panel B). The number achieved for trained items is given on the left vertical axis, and the number of achieved untrained items is given on the right, with the maximum value of each scale adjusted according to the total number of items for that word set condition. Visual analysis indicates the initial baselines for each intervention block were relatively stable with low or no variability for all participants. A positive treatment effect, that is, an increase in the number of trained items from word sets 1 and 2 achieving the target priorities compared to pre-intervention baseline counts, was seen for all participants for some intervention blocks. The magnitude of treatment effect is reported in Table 5 for protophone targets and Table 6 for speech movement targets. As an overall summary across participants and outcome measures, the mean Tau coefficient effect size for trained items in word set 1 (i.e., items trained throughout the intervention block) was 0.61 (*SD* = 0.27, range 0.22–1.0), a large effect size. Of those 15 Tau coefficients, five were statistically significant with large or very large effect size. The mean difference in percent for the word set 1 trained items between the post-intervention and pre-intervention baselines was positive and averaged 37% (*SD* = 24.9, range 6.7–75.0). The Tau coefficient also correlated strongly with the mean difference scores (*r* = 0.86, *p* < 0.001, *n* = 15), confirming larger effects during the intervention phase for trained word set 1 items tended to be associated with a higher post-intervention mean (see also Figure 2, Figure 3, Figure 4, Figure 5, Figure 6, Figure 7, Figure 8 and Figure 9). 

The mean Tau coefficient effect size for trained word set 2 items, also calculated across participants and outcome measures, was 0.51 (*SD* = 0.36, range −0.1–1.0), a moderate to large effect size, with seven out of 15 coefficients being statistically significant with a large or very large effect size. The pre to post-intervention mean differences in percent averaged 35% (*SD* = 24.4, range 0.0 to 75) for trained word set 2 items. The correlation between the Tau coefficient and the mean difference for the same items was positive and statistically significant (*r* = 0.57, *p* = 0.026, *n* = 15). 

There was some limited evidence of generalisation to untrained items with five out of 30 Tau coefficients, all from either block two or three, being statistically significant with a large or very large effect size. The average mean difference across word set 1 and 2 untrained items was 23% (SD = 25.2, range −6.67–100). No changes were recorded in the control goal for any participant.

**P1**. In block one, intervention targeted controlled phonation, whilst moving the jaw from closed to open (closant–vocant) and open to closed (vocant–closant) syllable shapes, with target words containing predominantly bilabials. A moderate treatment effect was recorded for the protophone targets and speech movements on trained word set 1. In addition, a large and significant treatment effect was also observed in protophone targets for trained words containing bilabials in word set 2. Block two recorded a moderate treatment effect on trained word set 1 with generalisation to the untrained word set (moderate effect), where intervention targeted the production of protophones requiring open–close (vowel–consonant), close–open (consonant–vowel). Furthermore, a moderate treatment effect was observed on speech movements for the word set 2 trained items, and a significant effect for the untrained word set 2 items, showing the targeted vowels contained rounded and retracted lip movements. Block three recorded the largest treatment effects for protophone targets in word sets 1 and 2 and for speech movement targets in trained word sets 1 for items containing labial-fricatives (e.g., /f/, /v/) and lingual sounds (e.g., /d/, /g/), with large or very large effect sizes. Overall, there was a trend for larger and more consistent effects in block three for P1 compared to block one and two, which indicates a possible cumulative response to intervention. 

**P2**. Treatment effects were observed in the intentional use of vocalisations and target protophones and speech movements across all three intervention blocks. Block one targeted the production of protophone vocalisations with communicative intent, thereby decreasing non-communicative vocalisations. The data for four intervention sessions are missing due to technical failure. Nonetheless, the data show a large treatment effect on word set 2. Furthermore, the child vocalisation count, automatically generated from the LENA DLP, revealed a significant treatment effect, with decreasing vocalisations recorded within the home environment (Tau = −1, z = −2.393, *p* = 0.017). Block two recorded a moderate treatment effect on speech movement in trained word sets 1 and 2, where the intervention targeted jaw transitions from open-to-closed and closed-to-open; however, performance was variable. These changes in motor-speech control coincided with a large treatment effect in the number of target protophones for word set 1 trained items, with evidence of closant–vocant and vocant–closant productions, not previously sampled with communicative intent. Block three recorded treatment effects consistent with block two (e.g., significant effect for trained items from word set 2 for both protophone and speech movement targets), and showed evidence of generalisation of target protophone production in untrained words (word set 1). Treatment effects were greater for word set 2 than word set 1 for protophone production and speech movement targets, with evidence of CV (i.e., consonant–vowel), VC and CVCV productions, the phonemes /m/, /b/, /d/, /h/ and low vowels (e.g., /a/), for words such as bubble, bye and more. 

**P3**. P3 participated in two intervention blocks. During block one, there was a moderate increase in the production of controlled phonation on single protophones in word set 1, with no controlled phonation evident during baseline. Jaw transitions from closed-to-open and closed-to-open were not produced with communicative intent. These treatment effects were not maintained during post-intervention baseline. In contrast to block one, block two reveals a very large and significant treatment effect on trained word sets 1 and 2, for both protophone and speech movement targets, and large significant effects on word sets 1 and 2 untrained items for speech movement target for CV, VC and CVC words containing bi-labials (/b/, /p/, /m), alveolars (t/d/ and the velar (/g/). Post intervention data show the treatment effects were maintained for word sets 1 and 2. 

## 4. Discussion

The purpose of this study was to investigate the implementation of an early intervention protocol specifically designed for very young children with communication impairment secondary to CP. Our primary outcome measures focused specifically on (a) increasing the complexity of infant vocalisations produced with communicative intent, and (b) the establishment of motor-speech movements that would support the development of a core oral vocabulary.

Intervention blocks were multi-modal and framed within a Dynamic Systems Theory perspective, that posits increasing complexity arises from the bi-directional interaction of the components of a complex system [37]. Consequently, intervention block one was designed to build social interaction, with tactile input used to build the associate map between perception and speech production for the target vocabulary. Blocks two and three targeted motor-speech control more directly, with intervention priorities based on building subsystem control, as assessed using the MSH. Tactile input was used to shape key speech movement patterns required for the production of the target vocabulary. Our findings are presented with consideration given to the intervention priorities across the intervention blocks.

### 4.1. Block One: Building the Social Routine and Enriching the Environment

Intervention block one focused on building the social routine and creating activity dependent sensorimotor experiences for the shaping of motor-speech control. Our data show moderate treatment effects were observed in block one for participants 1 and 3 on the trained word set, with limited generalisation to the untrained word set. Treatment effects were not observed for the control word set. These results are consistent with our hypothesis of minimal change during this block, but are encouraging given all participants were pre-linguistic and had yet to establish communicative intent in their vocalisations. 

Previous studies show that language learning is dependent on building social routines and parent responsiveness to interactions [36]; with the reciprocity of the interactions contributing to increased vocal complexity [33]. Numerous studies have further demonstrated the effectiveness of training parent–child interactions in children with language impairment [32]. In addition, Pennington et al. [64] have also reported improved parent–child interactions in children with motor impairment following participation in the ITTT Hanen Program^®^. Accordingly, the increasing complexity of protophone production observed in this intervention block, could be considered a result of the increased responsiveness to the facilitated social feedback loop [65].

However, the fact that parents of participants in this study had all completed the ITTT Hanen Program^®^ as an entry requirement would suggest that the parents already were responsive to their child’s communication signals. As such, the therapeutic effect cannot be solely attributed to ongoing parent–child interaction. We postulate the tactile input that was mapped to the target words during the social routines and activities, provided a scaffold on which to build a template for word learning, and primed the child for word production [66]. Whilst the role of tactile input in building an associative map to enhance receptiveness to building oral vocabulary has not been fully explored in very young children, there is increasing evidence to support the role of auditory–tactile input connected with speech articulation [66]. Vihman et al. [67] suggest infants acquire language by the “implicit tallying of repeatedly experienced regularities in sensory input” [67], p. 129, with sound-meaning links more likely to be established when the input is highly familiar.

Furthermore, the literature has also identified that tactile input may reduce cognitive load [68] with haptic guidance enhancing motor learning by developing anticipatory activities and enhancing the “user’s presence and cooperation” [69], p. 37. Neurophysiological studies in adults suggest that congruent multi-sensory tactile input reduces ambiguity through cross-modal congruency [70]. It is therefore possible that the auditory–tactile input focused the child’s attention to the motor-speech action, making them more meaningful [66]. This warrants further investigation, particularly given the well documented risk of attention and memory deficits in children with CP [71].

We further postulate the experience of input from treatment block one positively influenced what Saffran and Kirkham [72] refer to as “downstream learning”. That is, the pre-existing vocal routines established in block one may have provided a “low-cost” communicative environment on which to build more complex vocal productions [72]. Therefore, the multi-dimensional focus of the first intervention block that focused on building routines that promoted turn-taking and the anticipation of the targeted vocabulary, along with the tactile input that linked the cognitive–linguistic input with the targeted output, may have been foundational to building motor-speech control in the subsequent intervention blocks.

### 4.2. Block Two and Three: Facilitating Motor-Speech Control

All participants continued to demonstrate increased production of target protophones and change in speech movement patterns that reflect increasing protophone complexity, in treatment blocks two and three. There was a trend for larger effect sizes during subsequent intervention blocks compared to treatment block one. This suggests a greater magnitude of treatment effect was observed in the intervention blocks where tactile input was used to shape motor-speech production.

Neurophysiological studies have shown difficulty planning and executing motor end goals experienced by children with CP may arise from impaired neural oscillatory activity in the sensorimotor cortices [73] and altered somatosensory organisation [74]. Speech production and ultimately language learning is a perceptuo-motor experience [75] with the role of the somatosensory input in building complexity through the proprioceptive consequences of the child’s own production, gaining increasing attention. For example, Choi et al. [14] identified when the proprioceptive-kinaesthetic information of an infant’s vocal tract is constrained, speech perception is disrupted. Conversely, when supplemental multimodal information is provided during active vocal play (e.g., contact of the fingers or an object on the lips), vocal complexity is increased [76]. We, therefore, hypothesise the tactile input assisted participants in acquiring the speech movement representation [40] and this is consistent with the literature supporting the role of augmentative feedback in improving motor learning in children with CP [77]. 

The contribution of tactile input in inducing therapeutic neuroplasticity has been demonstrated by Fiori et al. [41] in older children presenting with a motor-speech disorder. Whilst based on a small sample size, Fiori et al. [41] provide preliminary data that suggest the coupling of specific sensory information with specific movements can lead to treatment induced neuroplasticity. They identified not only changes in motor-speech control in children with the motor-speech disorder, childhood apraxia of speech, but also identified changes in white matter microstructural properties. The role of tactile input in children, in improving motor-speech control for infants with CP, therefore, warrants further attention.

It is noted that participants responded differently across the study phases, with change in level of performance greater on word set 1 than word set 2 for most participants, thus reflecting the first intervention priority as based on the MSH, in this intervention phase. This may be related to treatment dosage, with the rate of training in word set 1 at times three times more than word set 2. However, this finding is also consistent with the previous findings reported by Ward et al. [38,58] in older children with CP. Their research tested and supported the hypothesis that changes in motor-speech control at one level of the MSH would facilitate changes at the subsequent level of the motor-speech hierarchy as a result of inter-articulator coupling. 

Participant data also showed variability within the intervention phases, across the intervention blocks. This is expected and consistent with the literature reporting younger children experience more variability than older children [78]. This is observed not only in the development of language in typically developing infants, with the order and emergence of milestones variable [79,80], but also considered a hallmark of motor development [81], with variability considered the ongoing search for a solution to the motor strategy required. 

Our focus on infant vocalisations challenges what Brady et al. [82] has reported to be the prevailing clinical practice of abandoning efforts to increase speech production in children who are at risk of severe communication impairment and likely to be users of augmentative and alternative communication. Increasingly, emphasis is being placed on the potential therapeutic role of targeting infant vocalisations in infants at predicted risk of communication impairment to mitigate the severity of impairment.

It is argued that by supporting oral communication, the very act of practice and effort builds a more robust memory of representation that shapes phonological memory for later language learning [75]. A clear demonstration of the mediating effect of early intervention is evident in the research directed at children born deaf but provided with early access to hearing through cochlear implants. Infants who receive implants at less than 12 months typically progress to first words without delay and continue to perform well on language measures at a later age [83]. In contrast, children who receive implants later than 12 months can show deficits in the acquisition of first words and continue to perform more poorly on language measures. 

Similar access to early intervention in the emergence of canonical vocalisations in infants at-risk of CP may mitigate the cascading consequences of underlying impairment to speech motor control. Notably, we found the participants in this study continued to demonstrate improvements in their expressive language skills, as measured on the REEL-3. This contrasts with the findings of Ward et al. [29] who reported a worsening developmental trajectory at 24 months of age. This finding lends further support to our conclusion that the intervention was responsible for bringing about therapeutic change. The findings of this study could therefore be used to inform a larger longitudinal study. 

The focus on oral vocalisations for this study was based on a strong research foundation that has identified the critical importance of vocal play in developing later language skills, with the expectation that “…intervening at the prelinguistic stage may alter a child’s trajectory for producing spoken words” [84], p. 203, as well as semantic processing [67]. Oral motor dysfunction affecting speech related movements of the jaw, lips and tongue is high in children with CP [85]. These impairments can have significant functional consequences on speech intelligibility and communication. Thus, if infants at risk of communication impairment associated with CP are afforded the opportunity to experience more complex vocal play, we may ameliorate secondary impairments.

## 5. Limitations

There are a number of limitations to consider with this study. The highest level of single-subject research design (SSRD) is a randomised n-of-1 design, which may include random assignment of participant to treatment or order of treatment administration, as well as extended baselines when responses are more variable. This standard, whilst desirable, was not able to be met. At the time of this study, two additional multisite research trials were in process within this clinical population and age group. However, we mitigated risks to internal validity through the minimum of three data points per phase, systematic manipulation and assessment of the dependent variables by more than one assessor, and replication with at least three demonstrations of the experimental effect [42,43]. 

SSRD research requires repeated measures that are standardised, sensitive to change, reliable and valid. Given SSRD has been heralded as a methodology with clinical relevance, outcome measures should also be feasible to administer. The coding of speech probes for this study, however, required phonetic transcription, manual coding of the protophones, and visual-perceptual analysis of speech movements. The analysis was therefore labor intensive, and this potentially limits the clinically feasibility of the intervention. 

Furthermore, the outcome measures of this study required the a priori compilation of trained and untrained word sets with equal numbers of items in each word set. However, in response to participant interest and motivation, or the fact that some items did not get trained as intended, some words moved from their assigned word set resulting in a different number of items in the trained and untrained conditions (see Appendix A). A greater number of items within a condition may result in more opportunities to show improvement. This potential bias in effect size from differences in item numbers when comparing the trained and untrained data should be taken into account. However, we do note that the imbalance in item numbers does not systematically favour either the trained or untrained item conditions across participants. 

Finally, whilst parents were provided with a LENA device to record the elicitation of the speech probes once per week during intervention block one, and two times per week during intervention blocks two and three, home practice was not monitored to track cumulative treatment intensity. 

## 6. Conclusions

Children with CP are at predictable risk of communication impairment with impaired speech production being the most common form [1,2,3]. Yet, to date the earliest reported interventions for children with CP is greater than 2 years of age, well after the critical period where infants are primed for learning the basic building blocks required for later language and speech development and perceptual narrowing has already taken place [9,86]. The lack of evidence-based interventions for young infants with communication impairment and neurodevelopmental disability is well recognised [10]. The dearth of research in this space, therefore, places young infants with CP at increased risk of communication impairment. Our data suggest the speech skills of young children with CP can be positively influenced through a multimodal intervention thus capitalising on early periods of plasticity, when language learning is most sensitive. 

## Figures and Tables

**Table 1:** Participant Characteristics at Study Onset.

Participant	P1	P2	P3
Age at Intervention Block One	15 months	15 months	16 months
Sex	M	M	F
Diagnosis	Spastic Quadraparesis	Dyskinetic	Spastic Hemiplegia
Gestational Age	Term	35 weeks	Term
Age of diagnosis	<6 months	<6 months	<6 months
GMFCS at 2 years	III	III	I
Hearing Status	WNL	Aided	WNL
Oral Pharyngeal Dysphagia	Oral	PEG	Oral
Epilepsy	No	No	Stable

**Table 2:** Communication Status as Measured Across the Study Phases.

	Age of Assessment							
	Participant 1			Participant 2			Participant 3	
	15	24	36	15	24	36	15	24
**CSBS DP**	SS (PR)	SS (PR)	[RS]	SS (PR) [RS]	SS (PR)	[RS]	SS (PR)	SS (PR)
Communication Composite	120 (91)	107 (68)	NA	82 (12)	79 (8)	NA	102 (55)	96 (39)
Cluster Scores				SS (PR) [RS]	SS (PR)	[RS]	SS (PR)	SS (PR)
Function	14 (91)	5 (5)	NA	9 (37)	7 (16)	NA	10 (50)	10 (50)
Gestural	10 (50)	5 (5)	NA	3 (1)	8 (25)	NA	10 (50)	8 (25)
Vocal	15 (98)	6 (9)	NA	8 (25)	3 (1)	NA	12 (75)	7 (16)
Consonants used [#different]	[8] (3)	[9] (4)	[12]	[2] (2)	[5] (3)	[5]	[4] (3)	[7] (3)
	/m/, /n/, /b/, /d/, /g/, /w/, /j/, /dz/	/m/, /n/, /ŋ/, /b/, /d/, /t/, /g/, /w/, /dz/	/m/, /n/, /ŋ/, /b/, /p/, /d/, /g/, /w/, /v/, /s/, /dz/, /ʃ/	/n/, /g/	/m/, /n/, /d/, /g/, /w/	/m/, /n/, /b/, /d/, /w/	/m/, /n/, /d/, /j/	/m/, /n/, /b/, /p/, /d/, /g/, /j/
Verbal	* 11 (63)	7 (16)	NA	7 (15)	6 (9)	NA	* 11 (63)	6 (9)
DW	[8]	[11]	[45]	[0]	[5]	[10]	[2]	[8]
DWC	[3]	[3]	[3]	[0]	[0]	[2]	[0]	[0]
Reciprocity	10 (50)	12 (63)	NA	8 (25)	8 (25)	NA	10 (50)	11 (63)
Social-affective signalling	14 (91)	13 (84)	NA	13 (84)	14 (91)	NA	10 (50)	13 (84)
								
**REEL-3**	SS (PR)	SS (PR)	SS (PR)	SS (PR)	SS (PR)	SS (PR)	SS (PR)	SS (PR)
Expressive	73 (3)	87 (19)	72 (3)	<55 (<1)	70 (2)	<55 (<1)	98 (45)	105 (63)
Receptive	81 (10)	82 (12)	108 (70)	<55 (<1)	<55 (<1)	85 (16)	72 (3)	78 (7)
Language Ability Score	109 (73)	81 (10)	88 (21)	<46 (<1)	54 (1)	64 (<1)	82 (12)	90 (25)

Note. CSBS DP = The Communication and Symbolic Behaviour Scales Developmental Profile; REEL-3 = Receptive-Expressive Emergent Language Test-3; SS = Standard Score; PR = percentile rank; RS = raw score; Function = Communication Function from CSBS; Gestural = Communication Means Gestural from CSBS; Vocal = Communication Means Vocal from CSBS; DW = Inventory of different words; DWC = Different word combination; NA = Not Applicable; * This standard score is reflective of a raw score of one word approximation.

**Table 3:** Intervention Priorities for each Participant across the Three Intervention Blocks.

Priority	Participant One		Participant Two		Participant Three
	**Block One**				
1	**Production of /m/, /b/ and /a/ with jaw transitions moving from closed to open (closant–vocant) and open to closed (vocant–closant) in target words, with controlled phonation.**		**Produce vocalisations with communicate intent, within a turn-taking routine (Decrease vocalisations without communicative intent)**		**Controlled phonation of the sounds /m/, /b/ and /a/ with communicate intent, in target words**
2	**Lip-to-lip contact producing bilabials in words that contain movements with broad lip rounding (e.g., moo, push, boo) or retraction (e.g., me, bee) in CV syllables**		**Increase complexity of vocalisations in target words as coded on SAEDV-R. Responsive vocalisations to include isolated continuant closant (m, b, a) or closant–vocants**		**Jaw transitions moving from closed to open and open to closed, producing consonant–vowel and vowel–consonant combinations**
3	Achieve tongue separation from jaw in production of the phonemes /n/, d/, /t/ in target words		Jaw transitions moving from closed to open and open to closed, with phonation		Broad lip rounding (e.g., moo, push, boo) or retraction (e.g., me, bee) in CV syllable structures
	**Block Two**				
1	**Jaw transitions moving from closed to open and open to closed, in syllables containing CV, CVCV, VC and CVC structures**		**Increase complexity of vocalisations in target words as coded on SAEDV-R with communicate intent (b, m, a) closant–vocant or vocant–closant (marginal babble)**		**Jaw transitions moving from closed to open and open to closed, producing consonant–vowel and vowel–consonant combinations**
2	**Broad lip rounding or retraction in CV syllable structures**		**Jaw transitions moving from closed to open and open to closed, producing targeted closant–vocant and vocant–closant combinations**		**Broad lip rounding (e.g., moo, push, boo) or retraction (e.g., me, bee) in CV syllable structures**
3	Tongue separation from jaw in production of the phonemes /n/, d/, /t/ in CVC, VCV and VC words		Produce anterior lingual sounds /d/, /n/ in target words		Separation of tongue from jaw in CV, VC and CVC syllable structures
	**Block Three**				
1	**Engage lower lip for production of fricatives /f/ and /v/**		**Increase complexity of vocalisations in target words as coded on SAEDV-R with communicate intent, CV, VC or VCV**		
2	**Tongue separation of jaw in production of the phonemes /n/, /d/, /s/**		**Jaw transitions moving from closed to open and open to closed, producing targeted consonant–vowel and vowel–consonant combinations**		
3	Sequenced movements over two syllables		Produce the anterior lingual sounds /d/, /n/ in target words		

Note. Bold font = targeted priorities 1 and 2. Priority 3 is a control goal and not targeted.

**Table 4:** Fidelity to Intervention Dosage.

	Block One			Block Two			Block Three	
	P1	P2	P3	P1	P2	P3	P1	P2
Teaching Episodes Per Minute	1.56	1.03	0.95	1.40	1.24	1.11	1.5	1.29
No of sessions attended	10	10	10	10	10	10	10	10
Active Therapy Duration	35.80	35.00	26.80	35.25	31.13	37.00	34	22.8
Proportion Word set 1/Word set 2	3.60	1.04	3.40	2.90	1.20	2.40	4.80	1.60

**Table 5:** Tau coefficient, z, *p* values and pre to post mean differences in percent for each word set for protophone scoring across Intervention Blocks.

	Participant 1					Participant 2					Participant 3			
	Tau	z	*p*	%*M*_diff_		Tau	z	*p*	%*M*_diff_		Tau	z	*p*	%*M*_diff_
Word set	Block One													
Word set 1 TR	0.43	1.10	0.272	6.67		0.22	0.52	0.606	20.00		0.40	1.13	0.26	16.67
Word set 1 UT	0.20	0.51	0.612	0.00		0.17	0.39	0.699	20.00		0.00	0.00	1.00	20.00
Word set 2 TR	**0.77**	**1.94**	**0.052**	40.00		**1.00**	**2.32**	**0.020**	25.00		0.00	0.00	1.00	10.42
Word set 2 UT	0.40	1.01	0.311	13.33		0.33	0.77	0.439	8.33		0.10	0.28	0.78	0.00
Word set 3 control	−0.33	−0.85	0.398	−6.67		0.00	0.00	1.000	0.00		0.00	0.00	1.00	0.00
														
	Block Two													
Word set 1 TR	0.43	1.10	0.272	42.86		**0.80**	**2.03**	**0.043**	33.33		**1.00**	**2.83**	**0.005**	68.06
Word set 1 UT	0.57	1.44	0.151	38.10		0.30	0.76	0.447	16.7		0.30	0.85	0.396	100.0
Word set 2 TR	0.20	0.51	0.612	33.33		0.60	1.52	0.128	11.11		**1.00**	**2.83**	**0.005**	73.61
Word set 2 UT	0.37	0.93	0.353	37.50		0.07	0.17	0.866	−6.67		0.35	0.99	0.322	63.33
Word set 3 control	−0.47	−1.18	0.237	0.00		0.00	0.00	1.000	0.00		0.00	0.00	1.000	6.67
														
	Block Three													
Word set 1 TR	**1**	**2.83**	**0.016**	75.00		0.37	0.93	0.353	20.00					
Word set 1 UT	**0.60**	**1.70**	**0.090**	65.00		**0.70**	**1.77**	**0.076**	26.67					
Word set 2 TR	**0.68**	**1.91**	**0.056**	75.00		**0.67**	**1.69**	**0.091**	40.00					
Word set 2 UT	0.25	0.71	0.480	15.00		0.50	1.27	0.205	16.67					
Word set 3 control	0.00	0.00	1.000	0.00		0.00	0.00	1.000	0.00					

Note. TR = trained, UT = Untrained. Bold font = statistically significant, *p* value < 0.1, where Tau was positive.

**Table 6:** Tau coefficient, z, *p* values, and pre to post mean difference in percent for each word sets for motor-speech movement patterns, scored across the Intervention Blocks.

	Participant 1					* Participant 2					Participant 3			
	Tau	z	*p*	%*M*_diff_		Tau	z	*p*	%*M*_diff_		Tau	z	*p*	%*M*_diff_
Word set	Block One													
Word set 1 TR	0.60	1.52	0.128	46.67							0.50	1.41	0.157	11.11
Word set 1 UT	−0.17	−0.42	0.673	−4.76							0.00	0.00	1.000	13.33
Word set 2 TR	−0.10	−0.25	0.800	26.67							0.00	0.00	1.000	0.00
Word set 2 UT	0.10	0.25	0.800	6.667							0.00	0.00	1.000	0.00
Word set 3 control	0.00	0.00	1.000	0.00							0.00	0.00	1.000	0.00
	Block Two													
Word set 1 TR	0.60	1.52	0.128	38.1		0.53	1.35	0.176	11.11		**0.98**	**2.76**	**0.006**	70.83
Word set 1 UT	**0.73**	**1.86**	**0.063**	42.86		0.20	0.51	0.612	0.00		**0.65**	**1.84**	**0.066**	66.67
Word set 2 TR	0.53	1.35	0.176	38.1		0.30	0.76	0.447	0.00		**0.80**	**2.26**	**0.024**	50.00
Word set 2 UT	0.03	0.08	0.933	8.333		0.00	0.00	1.000	0.00		**0.60**	**1.70**	**0.090**	40.00
Word set 3 control	0.00	0.00	1.000	5.556		0.00	0.00	1.000	0.00		0.00	0.00	1.000	0.00
	Block Three													
Word set 1 TR	**1**	**2.83**	**0.005**	75.00		0.30	0.76	0.447	20.00					
Word set 1 UT	0.28	0.78	0.437	45.00		0.23	0.59	0.554	20.00					
Word set 2 TR	0.55	1.55	0.120	68.75		**0.73**	**1.86**	**0.063**	33.33					
Word set 2 UT	0.60	1.70	0.090	10.00		0.40	1.01	0.311	16.67					
Word set 3 control	0.00	0.00	1.000	0.00		0.00	0.00	1.000	0.00					

Note. TR = trained, UT = Untrained. Bold font = statistically significant, *p* value < 0.1, where Tau was positive. * = Participant 2 block one motor-speech movements were not targeted.

## Data Availability

The data are not publicly available in accordance with consent provided by participants on the use of confidential data.

## Appendix A

The word-lists for each participant across the intervention blocks.

**P1**						
						
**Word set 1**			**Word set 2**			**Word set 3**
**Trained**	**Untrained**		**Trained**	**Untrained**		**Control**
**Block One**						
mama	mine		ball	boom		go
bu(bble)	bang		(peeka) boo	knee		dator (grandpa)
up	arm		more	bow		kaka (brother)
down	done		moo	pooh		push
out	oh oh		bee	pea		eat
	ta					
	Bye ^a^					
**Block Two**						
mine	pan		up	pull		go
yum	bag		pour	bow		dator (grandpa)
hot	hat		me	knee		kaka (brother)
out	arm		do	pooh		push
more	bang		bye	boom		eat
(bar)bie (BBQ)	one		here	pea		door
bubble	nigh nigh		done	two		
**Block Three**						
four	feet		dinner	allah		icecream
give	phone		mine	amen		marshmallow
sun	horse		dog	soup		hungry
have	knife		off	and		spaghetti
need	bought			sand		yoghurt
done						
^a^ A trained item that did not get trained, added to untrained data.						

**P2**						
						
**Word set 1**			**Word set 2**			**Word set 3**
**Trained**	**Untrained**		**Trained**	**Untrained**		**Control**
**Block One**						
up	bu(bble)		more	oh oh		here
ou(t)	mama		moo	poo-ie		down
ah	oh		ball	wee		dada
arm	boat		baa	boo		bang
done	bye					
						
**Block Two**						
ou(t)	done		baa	poo (i)		here
ah	oh		mama	oh oh		down
more	bye		moo	boo		dada
bu(bble)	boat			wee (i)		bang
up				Me ^a^		
arm ^b^						
						
**Block Three**						
ball	bowl		out	bird		boat
mama	hat		up	bee		down
open	bye		boo(k)	baby		put
mine	done		more	pull		shoe
dada	apple		bubble			
^a^ A trained item that did not get trained, added to untrained data. ^b^ An untrained item that did get trained, added to trained data.						

**P3**						
						
**Word set 1**			**Word set 2**			**Word set 3**
**Trained**	**Untrained**		**Trained**	**Untrained**		**Control**
**Block One**						
baa	bag		ball	pooh		out
bubble	paper		go	boo		cat
under	bye		arm	do		eat
up	hat		push	bee		dirty
more	done			me		nose
moo ^c^						
**Block Two**						
more	paper		bubble	pooh		push
up	bye		out	boo		cat
arm	baa		bag	do		nose
ball			me	bee		dirty
hat ^b^			go	moo		eat
done ^b^			under			
						
^b^ An untrained item that did get trained, added to trained data. ^c^ Moved from word set 2 trained to word set 1 trained.						
